# Coral kin aggregations exhibit mixed allogeneic reactions and enhanced fitness during early ontogeny

**DOI:** 10.1186/1471-2148-8-126

**Published:** 2008-04-30

**Authors:** Keren-Or Amar, Nanette E Chadwick, Baruch Rinkevich

**Affiliations:** 1 Israel Oceanographic and Limnological Research, Tel-Shikmona, P.O. Box 8030, Haifa 31080, Israel; 2 The Mina and Everard Goodman Faculty of Life Sciences, Bar-Ilan University, Ramat Gan 52900, Israel; 3 Department of Biological Sciences, 101 Rouse Life Sciences Building, Auburn University, Auburn, AL 36849, USA

## Abstract

**Background:**

Aggregated settlement of kin larvae in sessile marine invertebrates may result in a complex array of compatible and incompatible allogeneic responses within each assemblage. Each such aggregate can, therefore, be considered as a distinct self-organizing biological entity representing adaptations that have evolved to maximize the potential benefits of gregarious settlement. However, only sparse information exists on the selective forces and ecological consequences of allogeneic coalescence.

**Results:**

We studied the consequences of aggregated settlement of kin larvae of *Stylophora pistillata *(a Red Sea stony coral), under controlled laboratory settings. When spat came into contact, they either fused, establishing a chimera, or rejected one another. A one-year study on growth and survivorship of 544 settled *S. pistillata *genotypes revealed six types of biological entities: (1) Single genotypes (SG); (2) Bi-chimeras (BC); (3) Bi-rejecting genotypes (BR); (4) Tri-chimera entities (TC); (5) Three-rejecting genotypes (TR); and (6) Multi-partner entities (MP; consisting of 7.5 ± 2.6 partners). Analysis of allorecognition responses revealed an array of effector mechanisms: real tissue fusions, transitory fusions and six other histoincompatible reactions (borderline formation, sutures, overgrowth, bleaching, rejection, and partner death), disclosing unalike onsets of ontogeny and complex modes of appearance within each aggregate. Evaluations at the entity level revealed that MP entities were the largest, especially in the first two months (compared with SG: 571% in the first month and 162% in the seventh month). However, at the genotype level, the SG entities were the largest and the colonies with the highest-cost-per-genotype were the TR and the MP colonies. The cost was calculated as reduced average genotype size, from 27% and 12% in the first month to 67% and 64% in the seventh month, respectively. In general, MP exhibited the highest survivorship rate (85%, after one year) and SG the lowest (54%).

**Conclusion:**

In view of the above, we suggest that the driving force behind gregarious kin settlements in *Stylophora pistillata *stems from gained benefits associated with the immediate and long-term increase in total size of the MP entity, whereas survivorship rates did not draw a parallel link. Furthermore, the biological organization of MP entity exhibits, simultaneously, an intricate network of rejecting and fusible interactions in a single allogeneic intimate arena, where proposed benefits surpass costs incurred by discord among founders. Above results and documentations on gregarious settlement in other marine taxa bring us to suggest that the 'group level' of kin aggregates may serve as a ubiquitous legitimate selection entity in the evolution of a sessile mode of life in marine organisms.

## Background

Natural chimerism, the biological state in which an organismal entity comprises cell populations originating from more than a single distinct fertilization product [1], is documented in a wide range of organisms including protists, animals and plants [2-10]. However, it is commonly perceived that the reverse situation, cellular uni-clonality within a biological entity, is a key evolutionary tool for prevention of inner-organism conflict and cellular pervasiveness [11,12]. Although research on animal and plant chimerism dates back to the early twentieth century [13,14], it is surprising that only sparse information exists on the ecological costs and benefits of genetically non-homogeneous biological entities. On one hand, vertebrate and invertebrate chimeras have been found to carry significant costs. Studies on invertebrates showed reduced growth rates, morphological resorption and necrosis [6,8,15,16]. In vertebrates, autoimmune diseases, freemartins and other abnormal syndromes have been recorded [1]. The literature also documents the expression of somatic and germ cell parasitism in vertebrates and invertebrates alike [1,4,10,17-19]. Various experimental manipulations during vertebrate and invertebrate development have revealed natural chimerism, often at early stages of ontogeny [1,6,15,20]. This phenomenon is enhanced in some sessile marine invertebrates by the gregarious co-settlement of larvae [15,21,22]. The above outcomes raise a critical question: What selective forces favour natural chimerism, in which there are so many 'losers' and so much to lose?

Some studies on aggregated settlement and chimerism have attributed an assortment of advantages in hard and soft corals [5,15,22], sponges [23,24], bryozoans [25], hydrozoans [26], and ascidians [21]. These include the expression of heterosis, increased genetic repertoire, reduced onset of reproduction, increased competitive capabilities, enhanced growth and survivorship rates, synergistic complementation and assurance of mate location [4,27-34]. Similar conclusions followed the formation of differentiated multicellular slugs by aggregates of social amoebae [31].

Gregarious co-settlement of conspecific larvae [13,15,22,28] creates suitable conditions for the development of a complex array of both compatible and incompatible allogeneic responses, because the genotypes within each chimeric entity or the rejecting partners within an aggregate are engaged in several types of allogeneic encounters. It is therefore possible to consider an entire aggregate of co-settled larvae (and its multiple allogeneic responses) as a self-organizing distinct biological entity, and inquire if adaptations have evolved to maximize the potential benefits of their gregarious settlement.

Observations (B. Rinkevich, unpubl.) documented in the wild, frequent aggregated spat of the Red Sea branching coral *Stylophora pistillata*. In order to elucidate the costs and benefits (in terms of growth and survival) of various types of such assemblages, we examined in controlled laboratory settings, the outcomes of six different classes of aggregates of settled kin larvae and their alloimmune responses in young *S. pistillata *colonies for up to one year. Interactions among spat of *S. pistillata *revealed various levels of costs and benefits for mixed alloimmune contacts within each aggregate. We therefore conclude that the 'group level' of kin aggregates may act as a legitimate selection entity in sessile marine organisms that settle gregariously.

## Results

**Figure 1:**
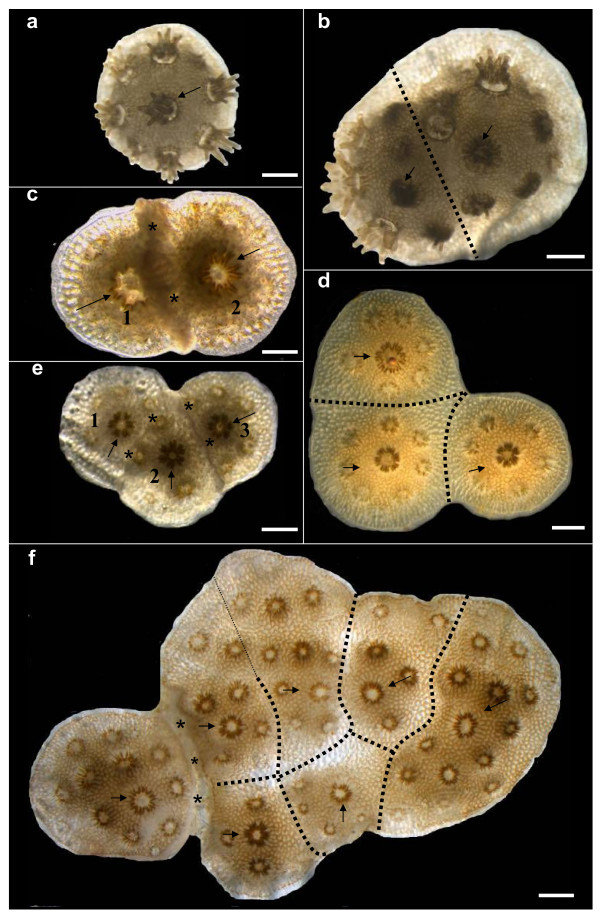
**Types of allorecognition entities in young colonies of *Stylophora pistillata***. (a) Single genotype (SG, age two months). (b) Bi-chimera (BC, age two months). (c) Bi-rejecting genotype (BR, age two weeks). Colony no. 2 has begun to overgrow colony no. 1. (d) Tri-chimera (TC, age 1.5 months). (e) Entity with three rejecting genotypes numbered 1, 2 and 3 (TR, age one month). Clear borderlines demarcate each genotype. (f) Aggregated colony composed of seven genotypes (MP = multi-partner entity, age four months). A necrosis developed between the left genotype and its two contacting confreres (out of the six fused partners), resulting in tissue separation. Asterisks indicate rejecting areas. Thick dotted lines depict presumed borders between genotypes. Thin dotted lines indicate no clear border between genotypes. Arrows point to the sites of founder polyps in each genotype. Scale bars = 1 mm.

When the coral larval recruits encountered each other, one of two alternate responses occurred. The spat either fused and established a chimera, or failed to fuse. The failure to fuse and its consequences was termed as rejection. Allogeneic incompatibility varied over time during ontogeny, with diverse responses following the initial non-fusion response (see below). In this study, the consequences of aggregated settlement of kin *Stylophora pistillata *larvae were investigated. Tissue fusion between allocompatible siblings developed within several days of settlement. Fused partners in BC and TC entities (n = 66 and 27 spats, respectively; Fig. 1b,d) remained connected to each other during the entire 7–12 months of observations, without any sign of tissue disconnection. However, in other cases of fusion (BR and TR, 8/126 interactions; Table 1), the initial state of tissue fusion between partners developed into rejection reactions, a phenomenon termed as transitory fusion ([6]; see below).

**Table 1:** Summary of the onset of allogeneic outcomes between young kin colonies of *Stylophora pistillata*.

Order of allogeneic mechanism	Total interactions		Number of allogeneic interactions that exhibited each mechanism of rejection (% of total interactions in each order) (% of total rejections in each order)						
	
	Fusions	Rejections	Transitory fusion	Borderline	Suture	Overgrowth	Bleaching	Necrosis	Death
First	59	65	8 (5)(14*)	52 (42)(80)	7 (6)(10)	6 (5)(9)	0	0	0
Second	51	61	0	3 (3)(5)	15 (13)(25)	9 (8)(15)	4 (4)(7)	28 (25)(46)	2 (2)(3)
Third	51	27	0	3 (3)(11)	4 (5)(15)	3 (4)(11)	1 (1)(4)	10 (13)(37)	6 (8)(22)
Fourth	51	12	0	1 (2)(8)	0	3 (5)(25)	0	7 (11)(58)	1 (2)(8)

* calculated out of fusions because at this stage the transitory reactions were observed as fusions.

**Figure 2:**
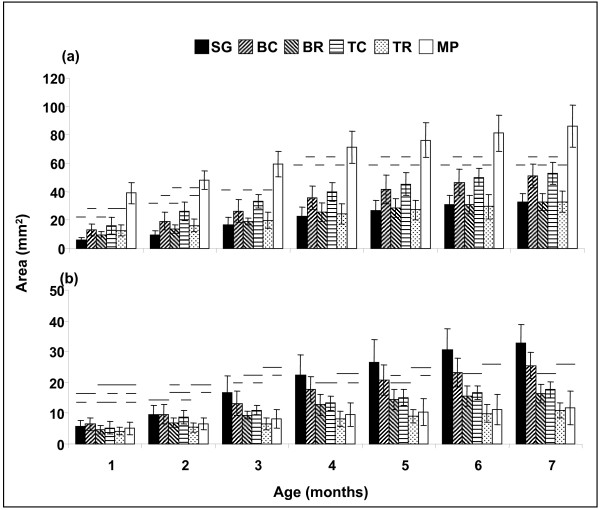
**Growth during the first seven months of young entities of *Stylophora pistillata***. Shown are areal sizes (a) per entity, and (b) per genotype (mean ± SE). Groups joined with the same line are not significantly different. Significant differences were at p < 0.05 as determined by one-way ANOVA followed by Duncan's post hoc tests. SG = single genotype, BC = Bi-chimera, BR = Bi-rejecting, TC = Tri-chimera, TR = Tri-rejecting, and MP = Multi-partner entity.

Growth was monitored at the entity level (Fig. 2a) and at the genotype level (Fig. 2b). During the entire observation period, the multi-partner (MP) entities were significantly larger at the entity level than all other five entity types (ANOVA, F = 14.0 followed by Duncan's post hoc test, p < 0.001, Fig. 2a). At the genotype level, after the age of two months, partners participating in the MP entities were smaller than partners in most other entity types (except the TR entity, Fig. 2b; ANOVA, F = 17.6, followed by Duncan's post hoc test, p < 0.001 at the age of 4 month and up). At the entity level, BC and TC were significantly larger than SG (Fig. 2a), whereas at the genotype level, SG colonies were significantly larger than BC and TC partners from the third month following settlement (Duncan's post hoc test, p < 0.001, Fig. 2b). Entity sizes of BC and TC did not differ significantly from each other (p > 0.05, Fig. 2a). However, after four months, the sizes of individual genotypes in the BC were larger than in TC (p < 0.001, Fig. 2b). BR and TR sizes at the entity level were similar (p > 0.05, Fig. 2a), but per genotype, BR were significantly larger than TR from the age of three months (p < 0.001, Fig. 2b). On the entity level, BR, TR and SG entities were the smallest of all examined entity types (Fig. 2a, b).

**Figure 3:**
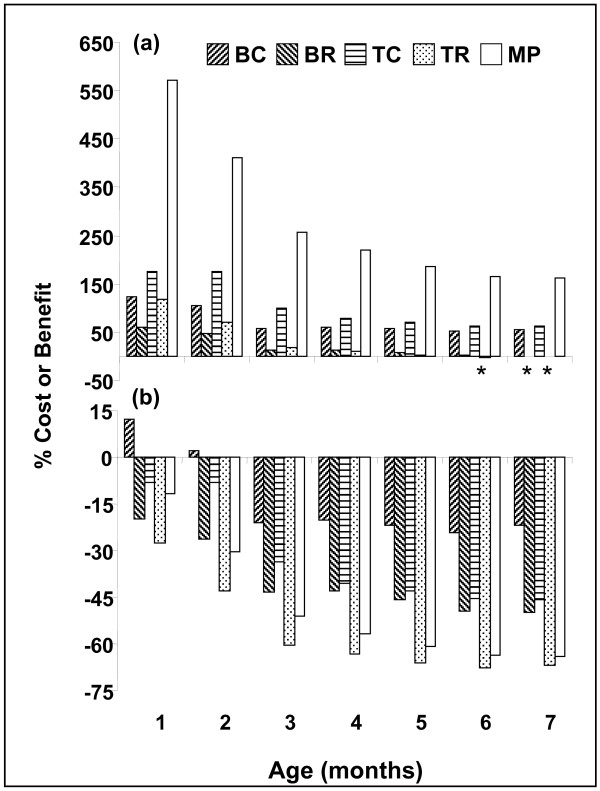
**Costs and benefits in terms of calculated areal size (in mm^2^)**. (a) per entity and (b) per genotype for BC, BR, TC, TR and MP entities, as compared to SG. Negative values refer to cost, positive values refer to benefit. Asterisks indicate small negative values. See Fig. 2 and text for explanation of abbreviations and sample sizes.

Quantitative costs and benefits in terms of growth were calculated at the entity level (Fig. 3a) and at the genotype level (Fig. 3b). At the entity level, MP colonies were the largest, and the benefits (measured as increased entity size) ranged from 571%, compared to SG in the first month, to 162% in the seventh month. Colonies that expressed histoincompatibility reactions (BR and TR) incurred a cost in terms of reduced entity size (Fig. 3a). At the genotype level, colonies with the highest cost per genotype were TR and MP. This cost (measured as reduced average genotype size) increased from 27% and 12% in the first month, to 67% and 64% in the seventh month, respectively (Fig. 3b). BC was the only entity type to show some benefit at the genotypic level (measured as increased average genotype size): in the first two months after settlement, each genotype benefited 12% and 2% (respectively) in size. However, at seven months, genotypes in the BC entities revealed a 22% cost in size (Fig. 3b).

**Figure 4:**
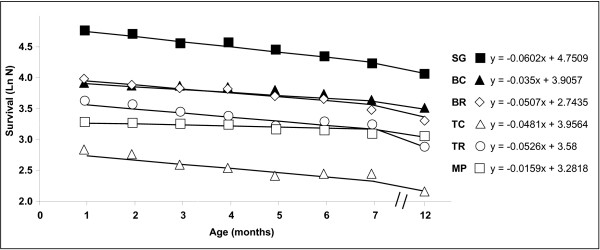
**Survival rates of six entity types of *Stylophora pistillata *(SG, BC, BR, TC, TR and MP) during the first 12 months of age**. N = number of survivors. See Fig. 2 and text for explanation of abbreviations and sample sizes.

Survival rate did not vary significantly among the six entity types (χ^2 ^= 0.11, df = 4, P > 0.05). However, regression analyses [35] revealed that MP colonies had the highest survival rate after 12 months with the most moderate slop (-0.016, Fig. 4), whereas the lowest survival rate was in SG with the sharpest slope (-0.060, Fig. 4). Intermediate survival rates were documented for BC, BR, TC and TR (-0.035, -0.051, -0.048 and -0.053 respectively).

**Figure 5:**
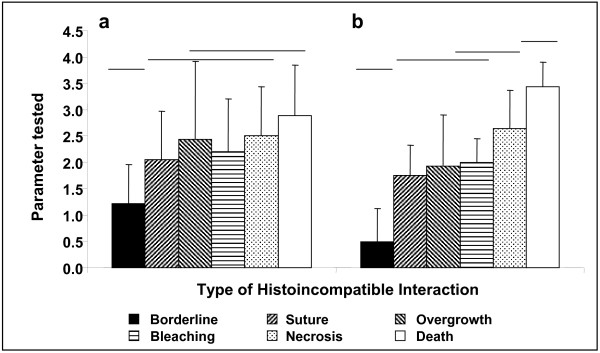
**Development of allorecognition interactions in *Stylophora pistillata***. a. Order of the incompatible interactions, and b. Age at which these responses were observed (mean ± SD). Groups joined with the same line are not significantly different (p > 0.05; One-way ANOVA followed by Duncan's post hoc tests). Sample sizes are detailed in Table 1.

A state of stable fusion was documented in 41% of interactions (51/124, Table 1). In 5% (of all interactions) or in 14% (of initially-observed fusions) of cases, the initial state of tissue fusion developed into transitory fusion [6,36], and revealed rejection reactions (Table 1). Half of the transitory fusions (4/8) changed into border line, and all developed into rejections around the second month after initial tissue contact (2.1 ± 1.1 months). Rejection reactions, comprising the six above types of incompatible interactions, developed in 53% of the cases directly after establishing of tissue-to-tissue contacts (Table 1). In 42% of total interactions (80% of rejections; Table 1), incompatible interactions started with the formation of a borderline. In general, this type of interaction appeared significantly earlier than the others (at an average order of 1.2 ± 0.6 and average age of 0.5 ± 0.9 months, ANOVA, F = 23.9, followed by Duncan's post hoc test, p < 0.001, Fig. 5). Then, the histoincompatible reactions developed into other morphological types. No single interaction began with necrosis or colony death. However, in most cases, interactions culminated in either tissue necrosis (at an average order of 2.5 ± 0.4, p > 0.05, Fig. 5), or in the death of one of the partners (at an average order of 2.9 ± 0.5, p > 0.05, Fig 4). Tissue necrosis (onset at 2.6 ± 0.9 months, p > 0.05, Fig. 5) and death of one of the partners (3.4 ± 1.0 months, ANOVA, F = 35.7, followed by Duncan's post hoc test, p < 0.001, Fig. 5) were the last effector mechanisms in the cascade of rejection events. Only 6% of all non-fusible cases (4/65) displayed a single histoincompatible response during the entire period of 12 months. However, in the development of rejection, 18% of non-fusion interactions (12/65) exhibited four different allogeneic responses. Expression of the final allogeneic interaction in each interacting pair was achieved at the age of 2.6 ± 1.2 months, with the formation of a fourth set of interactions (12 interactions, 7 of which were necrosis; Table 1). Most deaths occurred as the third alloincompatible reaction type (6/9; Table 1). We did not observe that tissue fusion was preceded by any type of allogeneic rejection.

## Conclusion

Organismal uniclonality is assumed to be beneficial in preventing inner-organism conflicts [11,12]. Therefore, the occurrence in nature of genetically non-homogeneous entities (chimeras) in a variety of protists, fungi, plants and animals is perplexing ([2-6,8,10,15] and literature therein). The occurrence of chimeras also challenges the paradigm for evolutionary benefits associated with genetically homogeneous multicellular organisms [7,9,10,37], the traditional notion of the unit of selection [7,37], and our perception of multicellular stability [33,34,38]. The scientific literature attributes many benefits to the state of chimerism [4,16,27-34], especially in cases of fusion between kin, rationalizing a chimera as a congruent entity [15]. However, if coral planulae are gregarious settlers, then they may fuse by virtue of physical proximity regardless of selective advantage. It is therefore natural to ask, is chimerism in the stony coral *Stylophora pistillata *evolutionarily beneficial? How shall we consider cases where, in addition to chimerism, a gregarious settlement may create groups of kin organisms, each exhibiting concurrently an intricate network of rejecting and fusible interactions?

Given the meagre knowledge on coral population genetics and the limited competency for genetic discrimination, the currently available molecular tools do not allow the evaluation of genetic relatedness in natural coalescences. We chose to analyse kin aggregates in a laboratory, where replicates can be generated with tens of planulae per Petri dish. Under our controlled laboratory conditions, *Stylophora pistillata *sibling larvae had a single ontogenic opportunity to choose whether to live in an aggregate. Most larvae (67%) selected the option of co-settlement and kin intimacy [22]. Each established kin aggregate of coral spat exhibited mixed allogeneic responses, from true tissue fusion to rejection, and ending in tissue necrosis and colony death. Such an aggregates mode of settlement, which leads to a variety of simultaneously-developing allogeneic encounters, is unlikely to occur unless selective pressure favours its existence [27]. Our study reveals that the common formation of multi-partner (MP) entities implicates the 'group level' (an assemblage of several genotypes, both rejecting and fusing, in one entity) as a key level at which natural selection may occur. Our results at the group level contradicted our genotype-level results. The mixed-allogeneic MP entity in this study (7.5 ± 2.6 partners) was, in its first month, 6.7 times larger than the single genotype (SG) entity. This consistently decreased to a ratio of only 2.6 at the age of seven months. On the genotype level, the areal sizes of SG colonies were 1.1 times larger than MP genotypes in the first month, and this trend increased consistently to 2.8 times at the age of seven months. We propose, therefore, that the benefit of being a partner in a MP entity (either engaged with rejection or fusion interactions) lays in the sensitive early phases of ontogeny, in which the ability to reach rapidly a large size and to occupy a large substrate space relative to other individuals is subject to intense selection pressure in sessile marine invertebrates (sensu; [4,27,28]). Successful long-term control of a feeding substrate by large MP entities of *Stylophora pistillata *effectively prevents colonization of this surface area by other competing taxa, and likely increases competitiveness toward existing neighbours. Since kin larvae form aggregates preferentially [22], the establishment of MP entities, while it minimizes space exploitation by each settling genotype, may increase the inclusive fitness of close relatives within each aggregate.

The maintenance of MP entities comes at a cost. Calculations made at the genotype level revealed a single genotype size cost ranging from 12% (in the first month) to 64% (in the seventh month). This was not the case with the smaller aggregates tested; the costs of maintenance of bi-chimeras (BC) and tri-chimeras (TC) at the genotype level were low compared to SG colonies, ranging from a benefit of 12% in the first month to a cost of 22% in the seventh month in BC colonies, and costs of 8%–46% in TC. These results contrast with known outcomes for BC and TC entities in colonial urochordates (*Botryllus schlosseri*, [16,39,40]).

There are numerous documented costs of genetically non-homogenous entities in sedentary marine organisms. These include reduced chimeric sizes in sponges [41] and soft corals [15], polyp/zooid resorptions in soft corals [15] and colonial tunicates [32,42], unstable entities in scleractinian corals [20], somatic and germ cell parasitism in botryllid ascidians [1,10,17,18] and decreased survivorship in tunicates [16,32,33]. However, when dealing with MP entities, laboratory studies on the tunicates *Botryllus schlosseri *[39] and *Botrylloides leachi *[40] revealed that multi-chimeras were more stable, grew faster, and had lower frequencies of colony resorption compared to bi-chimeras. In corals, multi-partner chimeras of *Pocillopora damicornis *were the largest entities among individual, bichimera and non-fused colonies, and with the highest survival rates [43], recalling our results here for survival. The evolutionary advantages of aggregated vs. solitary biological situations can be puzzling, and should be examined not only in the allorecognition context as depicted here, but also in other biological scenarios, such as the hatching success of solitary vs. aggregated nest events in marine turtles [44].

In conclusion, we reveal here that a driving force for gregarious kin entities, which develop by sedentary marine organisms like cnidarians [5,13,22] and urochordates [21], may stem from the benefits in terms of the total size of the aggregated entity, which includes rejecting and fusing partners. Therefore, in addition to previous studies suggesting that selection operates at the colony level [7], it is important also to consider the 'group level' of kin aggregates (revealing a network of historecognition responses) as a legitimate selection entity in the biology and ecology of sessile marine organisms. In such entities, intraspecific cooperation among kin may develop [10,27,39]. Explicit work should further consider arenas of non-related genotypes under laboratory conditions and when possible, *in situ *coalescences, where different genotypes simultaneously cooperate and compete under natural conditions.

## Methods

Planula larvae were collected *in situ *(March and April 2005; [6,22,45]) from 10 gravid colonies of *Stylophora pistillata *on the coral reef adjacent to the Interuniversity Institute for Marine Sciences (IUI, Eilat, Red Sea), and shipped to the laboratory at Israel Oceanographic and Limnological Research (IOLR, Haifa) within two days after collection. Planula settlement began within several hours post-collection and continued for up to three weeks. Groups of 50–70 planulae, each hatched from a single mother colony, were placed in polyester film lined, 60 mm Petri dishes, each containing 45 ml seawater (under these conditions the percentages of planulae that aggregate do not correlate with density; [22]). Under these conditions, 67% of the kin planulae settled in aggregations (spat <1 mm from each other) of at least two spat per aggregate, while the rest (33%) settled solitarily. Upon settlement, each individual spat or aggregated entity was numbered by pencil on the polyester film. Next, the surrounding film was trimmed, leaving each young colony on a small disc of film, which then was attached by cyanoacrylate glue (Super Glue 3, Loctite, Ireland) to a 5.0 × 7.5 cm glass slide. Detailed rearing methods for these young corals under laboratory conditions are described elsewhere [22,45]. In total, we monitored 544 metamorphosed larvae produced by 10 *S. pistillata *colonies.

First, we randomly selected 208 spat to measure growth, because it was logistically impossible to record the growth of all coral spat due to sampling time constraints. These spat were examined carefully each month for the next seven months. Spat were assigned to six entity classes according to the number of partners within each aggregation and their allorecognition response types: (1) Single genotypes (SG, n = 12, Fig. 1a); (2) Bi-chimeras, each consisting of two fused genotypes (BC, n = 11, Fig. 1b); (3) Bi-rejecting genotypes (BR, n = 11, Fig. 1c), (4) Tri-chimeras, each of three fused genotypes (TC, n = 7, Fig. 1d); (5) Tri-rejecting genotypes (TR, n = 16, Fig. 1e); and (6) Multi-partner entities that included both fusion and rejection patterns (MP, n = 11, Fig. 1f), each containing, at least, three fused partners (maximum number of 12 partners per aggregate, mean ± SD, 7.5 ± 2.6 partners, Fig. 1f). Costs and benefits were calculated for each of these six response types, in terms of growth in surface area, as a percent difference from the average surface area of SG entities, on the level of both whole entities and genotypes.

Next, we documented the survival rate of all 544 spat that settled both as individuals and in aggregates, each month for the first seven months and then again at the age of 12 months. Survival was determined for 113 SG, 48 BC, 51 BR, 16 TR, 34 TC, and 26 MP entities (n = 288 total entities and n = 544 total spat, since most entities contained >one coral spat). Each entity in the six classes containing more than a single genotype, was developed, from groups of planulae originated from a single mother colony. Data for spats from 10 maternal colonies in each class was pooled as no significant difference was recorded in the growth rates between the colonies within each class (One-way ANOVA, F = 1.3, p > 0.05) and survivorship of young spats was not affected by the mother origin (p > 0.05).

Then, we monitored the development of allorecognition in 223 randomly-selected *S. pistillata *spat for up to one year. These spat included some that were monitored for growth above, and some that were not. Four classes of entities were observed to determine the development of effector mechanisms: 33 BC, 35 BR, 9 TC and 20 TR. Multi-partner entities were not included in this experiment, because of the difficulty of monitoring simultaneously several different allogeneic interactions in each genotype. A state of true allogeneic fusion was recorded when fused partners remained continuously connected by intact tissue during the entire experimental period. A state of transitory fusion was recorded when partners initially appeared to undergo tissue fusion, but later developed an incompatible reaction. Six other morphological outcomes developed directly as incompatible effector mechanisms: (1) a borderline between contacting genotypes, without any sign of tissue necrosis; (2) suture formation [46] between partners, marked by a thin skeletal wall; (3) overgrowth, in which one genotype overgrew its confrere; (4) bleaching, in which a white region demarcated the interacting partners; (5) tissue necrosis followed by separation between the partners; and (6) death of one of the partners. We monitored the development of these allorecognition interactions over time. The order in which one transitioned to another was numbered, as well as the age at which each transition occurred.

Colonies were observed each week under a Nikon SMZ800 stereomicroscope. Photographs (once every two weeks during the first two months, and thereafter once per month) were taken with a Color View 2 Soft Imagin System camera equipped with a millimeter grid as a scale bar.

Data were analyzed statistically using SPSS software version 10 for Windows. Normality and homogeneity of variance were tested by Kolmogorov-Smirnov and Levene's statistical tests, respectively. ANOVAs followed by Duncan's post hoc tests were used for comparing the growth of the entity types, the ages at which the six allogeneic interactions occurred, and the order of development of allogeneic interactions. Chi square tests were used for evaluating survival rate. The results are presented as mean ± SD except where indicated.

## Authors' contributions

KOA and BR conceived and designed the experiments. KOA, NEC and BR wrote the paper. KOA performed the experiments, KOA, NEC and BR analyzed the data. BR and NEC contributed reagents/materials/analysis tools.
